# Association of weight-adjusted waist index with all-cause mortality among non-Asian individuals: a national population-based cohort study

**DOI:** 10.1186/s12937-024-00947-z

**Published:** 2024-06-12

**Authors:** Ting Cao, Ruijie Xie, Jiusong Wang, Meimei Xiao, Haiyang Wu, Xiaozhu Liu, Songlin Xie, Yanming Chen, Mingjiang Liu, Ya Zhang

**Affiliations:** 1 Department of Clinical Laboratory, The Affiliated Nanhua Hospital, Hengyang Medical School, University of South China, Hengyang, China; 2 Department of Hand & Microsurgery, The Affiliated Nanhua Hospital, Hengyang Medical School, University of South China, Hengyang, 421002 China; 3 Duke Molecular Physiology Institute, Duke University School of Medicine, Duke University, Durham, North Carolina USA; 4 Department of Cardiology, The Second Affiliated Hospital of Chongqing Medical University, Chongqing, China; 5 Department of Gland Surgery, The Affiliated Nanhua Hospital, Hengyang Medical School, University of South China, Hengyang, 421002 China; 6 The Affiliated Nanhua Hospital, Hengyang Medical School, University of South China, No.336 Dongfeng South Road, Zhuhui District, Hengyang, Hunan Province 421002 PR China

**Keywords:** All-cause mortality, Obesity, Weight-adjusted-waist, NHANES

## Abstract

**Introduction:**

The Weight-Adjusted Waist Index (WWI) is a new indicator of obesity that is associated with all-cause mortality in Asian populations. Our study aimed to investigate the linear and non-linear associations between WWI and all-cause mortality in non-Asian populations in the United States, and whether WWI was superior to traditional obesity indicators as a predictor of all-cause mortality.

**Methods:**

We conducted a cohort study using data from the 2011–2018 National Health and Nutrition Examination Survey (NHANES), involving 18,592 participants. We utilized Cox proportional hazard models to assess the association between WWI, BMI, WC, and the risk of all-cause mortality, and performed subgroup analyses and interaction tests. We also employed a receiver operating characteristics (ROC) curve study to evaluate the effectiveness of WWI, BMI, and WC in predicting all-cause mortality.

**Results:**

After adjusting for confounders, WWI, BMI, and WC were positively associated with all-cause mortality. The performance of WWI, BMI, and WC in predicting all-cause mortality yielded AUCs of 0.697, 0.524, and 0.562, respectively. The data also revealed a U-shaped relationship between WWI and all-cause mortality. Race and cancer modified the relationship between WWI and all-cause mortality, with the relationship being negatively correlated in African Americans and cancer patients.

**Conclusions:**

In non-Asian populations in the United States, there is a U-shaped relationship between WWI and all-cause mortality, and WWI outperforms BMI and WC as a predictor of all-cause mortality. These findings may contribute to a better understanding and prediction of the relationship between obesity and mortality, and provide support for effective obesity management strategies.

**Supplementary Information:**

The online version contains supplementary material available at 10.1186/s12937-024-00947-z.

## Introduction

Obesity is a complex metabolic disease [1]. Obesity has experienced a significant surge in prevalence over the decades and is reaching an unprecedented level: nearly one-third of the global population is obese [2, 3]. The condition has been unequivocally linked to mortality, and studies have estimated that obesity can reduce life expectancy by 5–20 years [4, 5]. The increased mortality risk is primarily attributable to the elevated incidence of chronic diseases, including cardiovascular disease [6, 7], metabolic disease [8, 9], Alzheimer’s disease [10], and various cancers [11–13], which are closely associated with obesity.

The Body mass index (BMI) is widely recognized as a reliable predictor of premature mortality, yet it fails to differentiate between fat mass and lean body mass [14]. Instead, body composition and distribution of body fat are more precise indicators of unfavorable metabolic profiles [15, 16]. It is worth noting that in a recent meta-analysis of 32 studies involving approximately 32,000 individuals with excessive body fat according to BMI, half of them were found to have normal body fat [17]. Thus, waist circumference (WC) has emerged as a potential alternative to BMI for forecasting obesity-related disorders [18]. However, WC and BMI are highly correlated, limiting WC’s independent use as a measure of BMI [19, 20].

The weight-adjusted waist circumference index (WWI), originally introduced by Park et al [21], provides a measure of age-related changes in body composition, including loss of muscle mass and retention or increase in fat mass [22]. Recent investigations demonstrate a noteworthy positive relationship between WWI and cardiovascular disease [23, 24], chronic kidney disease [25], and metabolic disease [26]. Moreover, prospective studies reveal that WWI is a reliable predictor of cardiac-caused mortality and all-cause mortality [21, 27, 28]. Nevertheless, these studies have solely focused on Asian populations. Additionally, the non-linear association between WWI and all-cause mortality requires further explanation, which is of paramount importance for effective obesity management aimed at reducing mortality.

Therefore, we conducted a cohort study using data from the 2011–2018 National Health and Nutrition Examination Survey (NHANES) to investigate the linear and non-linear associations between WWI and all-cause mortality in non-Asian populations in the United States, and whether WWI was superior to traditional obesity indicators as a predictor of all-cause mortality.

## Methods

### Study population

**Fig. 1:**
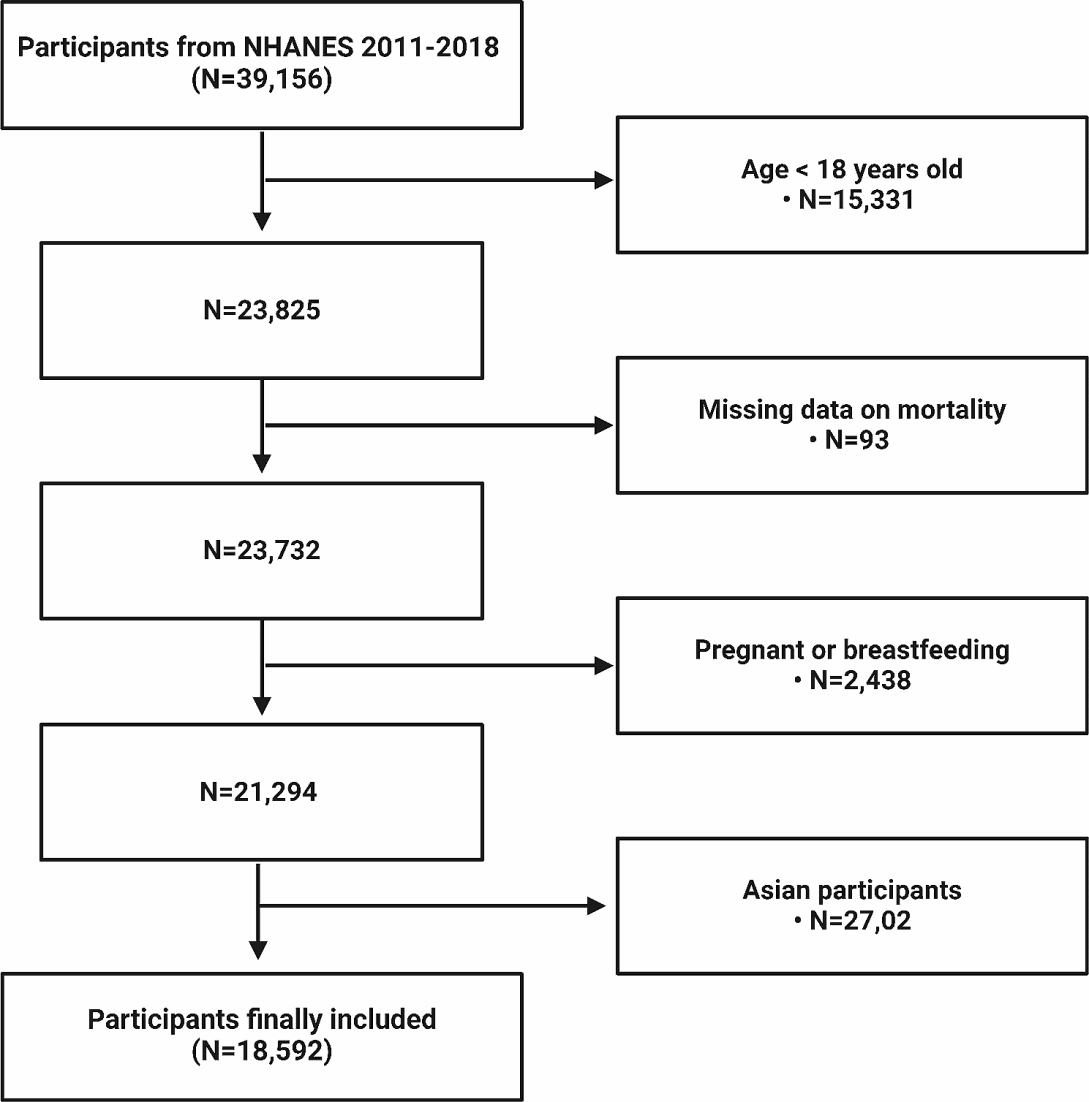
Flow chart of participants selection. NHANES, National Health and Nutrition Examination Survey

The NHANES is a widely recognized, nationally representative survey program administered by the National Center for Health Statistics (NCHS) [29, 30]. The NCHS Research Ethics Review Board approved the study procedure, and all participants provided written consent at the time of recruitment. To ensure data quality and reliability, we excluded 15,424 participants who lacked available mortality data or younger than 18 years, 1,294 participants with missing weight data, 1,122 participants without WC data, and 2,724 Asian participants. The final sample size, comprising individuals aged between 18 and 80 years, included 18,592 participants, as illustrated in Fig. 1.

### Weight-adjusted-waist index

WWI is calculated by dividing waist circumference (cm) by the square root of body weight (kg) [31]. At the mobile examination center, certified health technicians measured participants’ weight and waist circumference. The participants’ weights were determined by removing shoes and heavy clothing. In order to assess waist circumference, skilled health technicians employed a standardized protocol, which involved drawing a horizontal line above the right iliac bone’s highest lateral border and locating the right mid-axillary line. The intersection point of these two lines was marked, and a tape measure was precisely positioned to obtain an accurate measurement [32].

### All-cause mortality

To ascertain all-cause mortality, we consulted the records of the National Death Index (NDI) and linked them to the NHANES datasets [33]. Participants were included in the research from the date of their survey participation until either their death or 31 December 2019.

### Covariables

Based on previous studies of the association between body composition and mortality [34, 35], we included a variety of covariates that could potentially influence the outcome. These included age (as a continuous variable), gender (male or female), race (categorized into non-Hispanic White, non-Hispanic Black, Mexican American, and other races, the other races includes Multi-Racial individuals), smoking status (current smoker, former smoker, never smoked), alcohol drinking status (current drinker, former drinker, non-drinker), income-to-poverty ratio (PIR) (a continuous variable), and multiple disease conditions (including self-reported heart failure, coronary heart disease, hypertension, renal failure, liver conditions, diabetes, stroke, heart attack, and cancer). Physical measurements were obtained through medical examinations at mobile screening centers, while demographic variables, lifestyle habits, and disease conditions were collected by trained personnel through standardized documentation.

### Statistical analysis

All statistical analyzes were weighted in accordance with the NHANES analysis guidelines, given that NHANES employs a complex multi-stage probability sampling design [36, 37]. Due to the presence of missing data for several covariates, multiple imputation (5 datasets) with chained equations was performed to address this issue. All statistical analyses were performed using R (version 4.2) or Empowerstats (version 5.0). The demographics of the participants were assessed by quartile of the WWI using chi-square and t-tests. Cox proportional hazard models were employed to evaluate the association between WWI, BMI, WC and the risk of all-cause mortality. Three models were developed to control for potential confounders, Model 1 was unadjusted, Model 2 adjusted for gender, age, and race, and Model 3 adjusted for age, gender, race, education level, ratio of family income to poverty, alcohol drinking status, smoking status, and diseases status. Additionally, we performed tests for linear trend by entering the median value of each quartile of WWI as a continuous variable in the models. The same statistical analysis was subsequently performed in two sensitivity analyzes in which we excluded participants with major cardiovascular disease (myocardial infarction, coronary heart disease, and stroke) and cancer, and in a second sensitivity analysis in which we excluded participants with less than two years of follow-up. Subgroup analyses and interaction tests were performed to explore the link between WWI and all-cause mortality in populations of differing genders, races, ages, and disease statuses. Smoothing curve fitting was employed to examine the nonlinear association between WWI and all-cause mortality, and a two-stage Cox proportional hazard regression model was constructed based on the inflection point [38, 39]. The effectiveness of WWI, BMI, and WC in predicting all-cause mortality was evaluated using a receiver operating characteristics (ROC) curve study. The area under the ROC curve (AUC) with associated 95% CI for sensitivity and specificity is displayed. Statistical significance was defined as a two-sided *P*-value less than 0.05.

## Results

### Baseline characteristics

The study included 18,592 eligible participants, consisting of 9,080 men (48.84%) and 9,512 women (51.16%), with a mean (SD) age of 47.99 (18.52) years. The racial composition of the cohort was as follows: 7,738 non-Hispanic whites (41.62%), 4,844 non-Hispanic blacks (26.05%), 2,975 Mexican Americans (16.00%), and 3,035 participants belonging to other races (16.32%). The participants had a mean (SD) WWI of 11.07 (0.89) cm/√kg. The median follow-up time was 58 months, during which 1,065 deaths occurred, resulting in a cumulative incidence of death during follow-up of 5.73%. Participants in the highest quartile of WWI were more likely to be female, non-Hispanic White people and Mexican American, and older compared to those in the lowest quartile. Moreover, individuals with higher WWI exhibited lower levels of education and income, indicating a lower socioeconomic status. Additionally, higher WWI were associated with a higher prevalence of cardiovascular disease, renal failure, liver diseases, and cancer (Table 1).

**Table 1:** Basic characteristics of participants by weight-adjusted-waist index among U.S. adults

Characteristics	Missing, %	Weight-adjusted-waist index			
		Q1 (*n* = 4,648)	Q2 (*n* = 4,648)	Q3 (*n* = 4,648)	Q4 (*n* = 4,648)
Age (years)	0	35.34 ± 13.83	45.59 ± 15.45	51.42 ± 16.35	57.06 ± 16.66
Family PIR	9.2	3.03 ± 1.69	3.14 ± 1.65	2.96 ± 1.67	2.59 ± 1.58
Gender, (%)	0				
Male		60.11	52.04	46.87	32.78
Female		39.89	47.96	53.13	67.22
Race/ethnicity, (%)	0				
Non-Hispanic White		67.91	68.31	66.73	70.21
Non-Hispanic Black		15.92	11.08	10.52	9.47
Mexican American		6.27	9.57	11.54	10.76
Other races		9.90	11.04	11.20	9.56
Education level, (%)	5.3				
< high school		9.34	12.36	16.46	19.27
High school		18.92	21.57	25.17	26.94
> high school		71.74	66.08	58.37	53.79
Drinking alcohol, (%)	8.0				
Current		57.71	57.72	57.53	55.42
Former		16.99	20.03	21.01	18.51
Never		25.30	22.75	22.46	26.07
Smoking status, (%)	1.4				
Current		20.96	20.70	19.04	18.04
Former		14.94	23.50	29.11	30.85
Never		64.00	55.80	51.85	51.11
Diabetes, (%)	0.1				
Yes		2.78	7.95	14.58	26.55
No		97.22	92.05	85.42	73.45
Stroke, (%)	5.3				
Yes		0.78	2.04	3.06	5.47
No		99.22	97.96	96.94	94.53
Coronary heart disease, (%)	5.5				
Yes		0.89	2.16	4.31	7.54
No		99.11	97.84	95.69	92.46
Renal failure, (%)	5.3				
Yes		1.27	2.26	2.46	5.44
No		98.73	97.74	97.54	94.56
Hypertension, (%)	0.1				
Yes		12.80	28.61	38.96	52.36
No		87.20	72.39	61.04	47.64
Myocardial infarction, (%)	5.3				
Yes		0.59	2.20	4.59	7.54
No		99.41	97.80	95.41	92.46
Cancer, (%)	5.3				
Yes		5.10	10.19	11.84	17.33
No		94.90	89.81	88.16	82.67
Liver diseases, (%)	5.4				
Yes		5.10	10.19	11.84	17.33
No		94.90	89.81	88.16	82.67
Weight (kg)	0	74.57 ± 16.80	82.50 ± 19.32	87.36 ± 21.73	92.74 ± 24.93
Waist circumference (cm)	0	85.53 ± 10.55	97.28 ± 11.58	105.45 ± 13.08	116.18 ± 15.86
BMI (kg/m^2^)	0	24.82 ± 4.70	28.36 ± 5.27	30.93 ± 6.40	34.53 ± 7.73
WWI (cm/√kg)	0	9.95 ± 0.39	10.75 ± 0.17	11.33 ± 0.17	12.14 ± 0.41

Mean ± SD for continuous variables: the P value was calculated by the weighted linear regression model; (%) for categorical variables: the P value was calculated by the weighted chi-square test. Abbreviation: PIR, the ratio of income to poverty, BMI, body mass index; Q, quartile; WWI, weight-adjusted-waist index

### Association between WWI, BMI, WC and all-cause mortality

Weighted cox proportional hazards regression analysis showed that WWI was positively correlated with all-cause mortality, and both BMI and WC were negatively correlated with all-cause mortality (Table 2). In the fully adjusted model, each unit increase in WWI was associated with a 13% increased risk of all-cause mortality [HR = 1.13, 95% CI: (1.03, 1.25)]. Participants with a higher WWI (quartile 4) had a 14% higher all-cause mortality rate than those with a lower WWI (quartile 1), although this linear association was not significant. Both BMI and WC were inversely associated with all-cause mortality. Participants with higher BMI had a 34% reduction in all-cause mortality [HR = 0.66, 95% CI: (0.55, 0.80)] compared with those with lower BMI, and participants with higher WC had all-cause mortality compared with those with lower rate decreased by 27% [HR = 0.73, 95% CI: (0.60, 0.88)]. After excluding participants with major cardiovascular disease and cancer, the positive association of WWI with all-cause mortality remained [HR = 1.27, 95% CI: (1.12, 1.44)], whereas the association between BMI and WC with mortality was not statistically significant (Table S1). In another sensitivity analysis, in which participants with less than two years of follow-up were excluded, very similar results were obtained, and the positive association between WWI and all-cause mortality remained robust [HR = 1.18, 95% CI: (1.06, 1.33)], the associations between BMI and WC and all-cause mortality were not maintained (Table S2). In addition, given that mortality increases exponentially with age, we used age (< 40, 40–60, > 60) as a categorical variable in the model adjustment, and the results were consistent with the previous ones, with the significant negative association between WWI and all-cause mortality remaining (Table S3).

**Table 2:** Association between WWI, BMI, WC, and all-cause mortality

Exposure	number of participants/ number of events	Person-Years (years)	Model 1 HR (95% CI)	Model 2 HR (95% CI)	Model 3 HR (95% CI)
WWI (continuous)	1,065/18,592	90311.67	2.04 (1.90, 2.18)	1.28 (1.17, 1.39)	1.13 (1.03, 1.25)
WWI (quartile)					
Q1	94/4,648	23937.33	Reference	Reference	Reference
Q2	191/4,648	23028.08	2.13 (1.66, 2.72)	0.97 (0.76, 1.25)	0.97 (0.75, 1.26)
Q3	303/4,648	22280.33	3.51 (2.79, 4.43)	1.08 (0.85, 1.38)	0.97 (0.75, 1.25)
Q4	477/4,648	21065.92	5.93 (4.75, 7.40)	1.39 (1.09, 1.77)	1.14 (0.88, 1.48)
P for trend			< 0.001	< 0.001	0.092
BMI (continuous)	1,065/18,592	90311.67	0.98 (0.97, 0.99)	0.99 (0.98, 1.00)	0.98 (0.97, 0.99)
BMI (quartile)					
Q1	310/4,605	23465.08	Reference	Reference	Reference
Q2	273/4,641	22805.42	0.89 (0.75, 1.04)	0.64 (0.54, 0.75)	0.61 (0.51, 0.72)
Q3	258/4,637	22320.25	0.85 (0.72, 1.00)	0.67 (0.56, 0.79)	0.62 (0.52, 0.74)
Q4	214/4,689	21645.08	0.73 (0.61, 0.87)	0.76 (0.64, 0.91)	0.66 (0.55, 0.80)
P for trend			< 0.001	0.009	< 0.001
WC (continuous)	1,065/18,592	90311.67	1.01 (1.01, 1.01)	1.00 (0.99, 1.00)	0.99 (0.99, 1.00)
WC (quartile)					
Q1	207/4,640	23672.17	Reference	Reference	Reference
Q2	257/4,654	22812.75	1.27 (1.06, 1.53)	0.71 (0.59, 0.86)	0.74 (0.61, 0.90)
Q3	294/4,632	22208.67	1.51 (1.26, 1.80)	0.66 (0.55, 0.79)	0.64 (0.53, 0.77)
Q4	307/4,666	21618.08	1.62 (1.36, 1.93)	0.82 (0.69, 0.98)	0.73 (0.60, 0.88)
P for trend			< 0.001	0.180	0.006

Model 1: No covariates were adjusted. Model 2: Age, gender, race were adjusted. Model 3: Age, gender, race, education level, ratio of family income to poverty, alcohol drinking status, smoking status, stroke status, coronary heart disease status, renal failure status, liver diseases status, myocardial infarction status, and cancer status were adjusted

*Abbreviation* Q, quartile; WWI, weight-adjusted-waist index; BMI, body mass index; WC, waist circumference

**Fig. 2:**
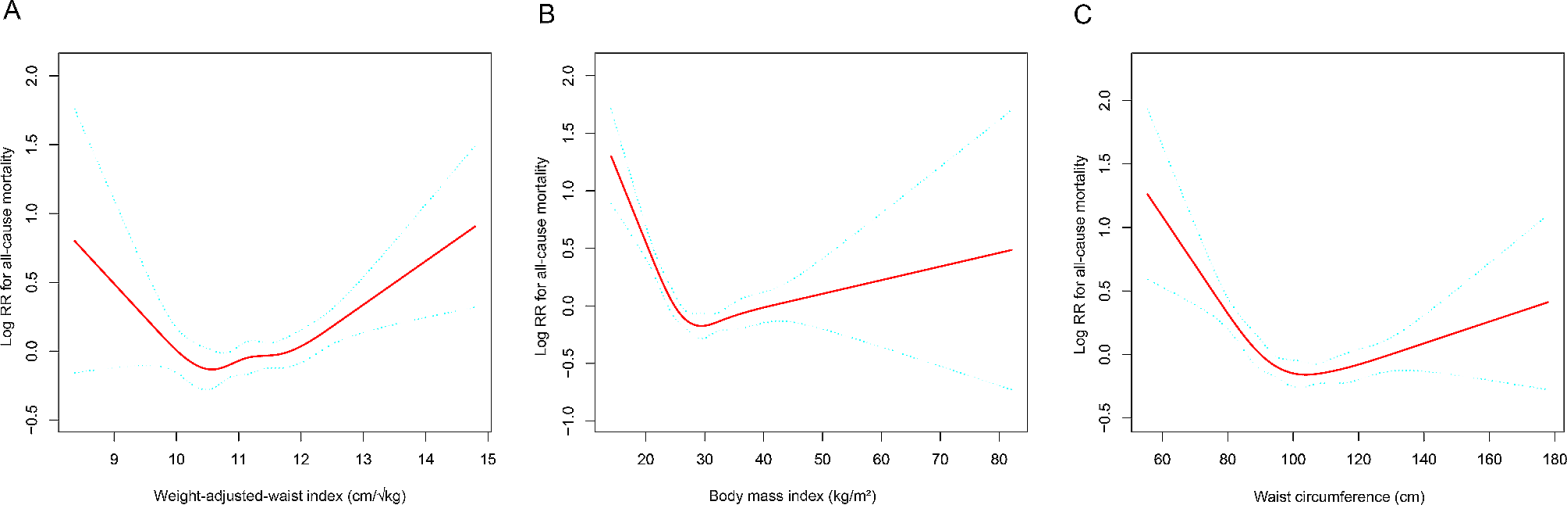
The nonlinear associations between weight-adjusted waist index, body mass index, waist circumference and all-cause mortality. The solid red line represents the smooth curve fit between variables. Blue bands represent the 95% of confidence interval from the fit. (**A**) weight-adjusted waist index and all cause mortality; (**B**) body mass index and all cause mortality; (**C**) waist circumference and all cause mortality

The smooth curve fitting results showed that there was a U-shaped association between the three obesity indicators and all-cause mortality (WWI: 10.46 cm/√kg; BMI: 28.50 kg/m^2^; WC: 101.40 cm) (Fig. 2). The results of the threshold effect analysis illustrate the inflection point (the lowest point of the U-shaped association) and the linear effects at both ends (Table 3). When WWI was greater than 10.46 cm/√kg, every unit increase in WWI was associated with a 20% increase in all-cause mortality [HR = 1.20, 95% CI: (1.08, 1.33)], while when WWI was less than 10.46 cm/√kg, each unit increase in WWI was associated with a 38% reduction in all-cause mortality [HR = 0.62, 95% CI: (0.41, 0.94)] (Table 3).

**Table 3:** Threshold effect analysis of WWI, BMI, and WC on all-cause mortality by two-piecewise linear regression model

Variables	number of participants/ number of events	Person-Years (years)	Adjusted HR (95%CI), P-value
Fitting by the standard linear model **(WWI)**	1,065/18,592	90311.67	1.13 (1.03, 1.25)
Fitting by the two-piecewise linear model			
Inflection point (cm/√kg)			10.46
WWI < 10.46 (cm/√kg)	94/4,621	23816.83	0.62 (0.41, 0.94)
WWI > 10.46 (cm/√kg)	971/13,971	66494.83	1.20 (1.08, 1.33)
Log likelihood ratio			0.007
Fitting by the standard linear model **(BMI)**	1,065/18,592	90311.67	0.98 (0.97, 0.99)
Fitting by the two-piecewise linear model			
Inflection point (kg/m^2^)			28.50
BMI < 28.50 (kg/m^2^)	583/9,246	45673.00	0.92 (0.90, 0.94)
BMI > 28.50 (kg/m^2^)	472/9,326	44562.83	1.02 (1.00, 1.03)
Log likelihood ratio			< 0.001
Fitting by the standard linear model **(WC)**	1,065/18,592	90311.67	0.99 (0.99, 1.00)
Fitting by the two-piecewise linear model			
Inflection point (cm)			101.40
WC < 101.40 (cm)	528/10,233	50935.00	0.98 (0.97, 0.98)
WC > 101.40 (cm)	537/8,359	39376.67	1.01 (1.00, 1.01)
Log likelihood ratio			< 0.001

Age, gender, race, education level, ratio of family income to poverty, alcohol drinking status, smoking status, stroke status, coronary heart disease status, renal failure status, liver diseases status, myocardial infarction status, and cancer status were adjusted

*Abbreviation* Q, quartile; WWI, weight-adjusted-waist index; BMI, body mass index; WC, waist circumference

**Fig. 3:**
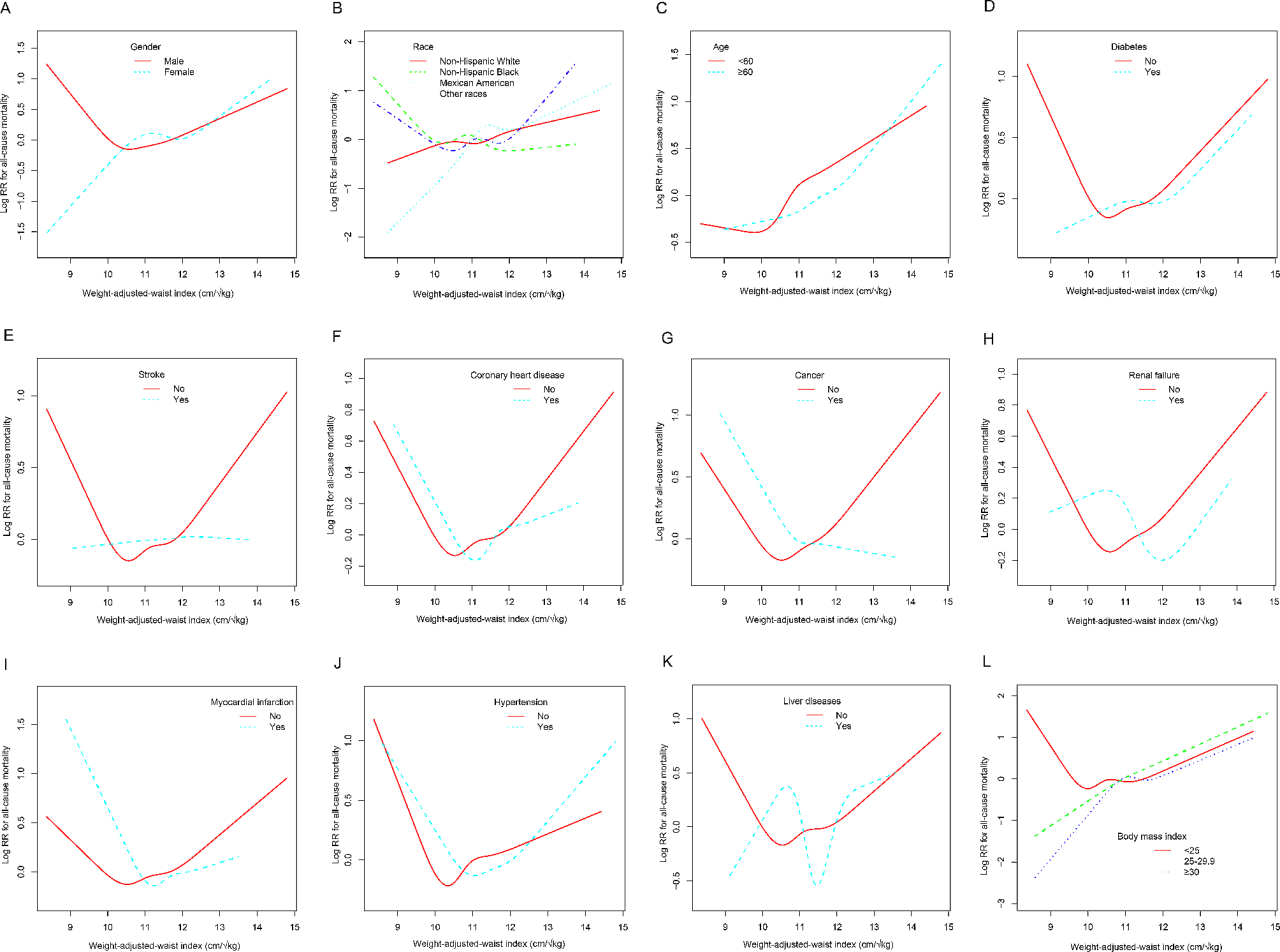
Subgroup non-linear relationship between weight-adjusted waist index and all-cause mortality. (**A**) Stratified by gender; (**B**) Stratified by race; (**C**) Stratified by age; (**D**) Stratified by diabetes; (**E**) Stratified by stroke; (**F**) Stratified by coronary heart disease; (**G**) Stratified by cancer; (**H**) Stratified by renal failure; (**I**) Stratified by myocardial infarction; (**J**) Stratified by hypertension; (**K**) Stratified by liver diseases; (**L**) Stratified by body mass index

To assess whether the association between WWI and all-cause mortality was consistent across populations, subgroup analyzes and interaction tests stratified by age, sex, race, and disease status were performed. The results in Table 4 show that race and cancer modified the association between WWI and all-cause mortality (interaction *P* < 0.05), and this association was even among non-Hispanic black people [HR = 0.87, 95% CI: (0.72, 1.05)] and cancer participants [HR = 0.83, 95% CI: (0.69, 1.01)] were negatively correlated. The results of smooth curve fitting confirmed these non-linear negative associations (Fig. 3). However, the association between WWI and all-cause mortality remained consistent across age, sex, and other disease subgroups (*P* > 0.05 for interaction).

**Table 4:** Subgroup analysis of the association between weight-adjusted-waist index and all-cause mortality

Subgroup	number of participants/ number of events	Person-Years (years)	Adjusted HR (95%CI)	P for interaction
Gender				0.380
Male	632/9,080	43940.42	1.10 (0.97, 1.24)	
Female	433/9,512	46371.25	1.19 (1.04, 1.35)	
Race/ethnicity				0.010
Non-Hispanic White	606/7,738	37867.50	1.21 (1.07, 1.37)	
Non-Hispanic Black	258/4,844	23902.17	0.87 (0.72, 1.05)	
Mexican American	92/2,975	14063.42	1.32 (0.96, 1.82)	
Other races	109/3,035	14478.58	1.44 (1.03, 2.03)	
Age				0.931
< 60 years	263/13,051	65725.00	1.35 (1.14, 1.59)	
≥ 60 years	802/5,541	24586.67	1.34 (1.20, 1.49)	
Diabetes				0.842
Yes	350/2,898	13050.25	1.14 (1.02, 1.28)	
No	715/15,694	77261.42	1.12 (0.95, 1.32)	
Stroke				0.393
Yes	141/693	2895.58	1.15 (1.04, 1.27)	
No	924/17,899	87416.08	1.02 (0.78, 1.32)	
Coronary heart disease				0.703
Yes	167/725	2958.75	1.13 (1.02, 1.26)	
No	898/17,867	87352.92	1.08 (0.86, 1.36)	
Renal failure				0.075
Yes	96/629	2798.00	1.16 (1.05, 1.28)	
No	969/17,963	87513.67	0.87 (0.65, 1.17)	
Hypertension				0.821
Yes	702/6,714	31562.75	1.09 (0.93, 1.28)	
No	363/11,878	58748.92	1.12 (0.99, 1.26)	
Myocardial infarction				0.136
Yes	168/758	3143.00	1.16 (1.04, 1.28)	
No	897/17,834	87168.67	0.95 (0.75, 1.21)	
Cancer				0.001
Yes	279/1,783	7888.42	1.23 (1.11, 1.37)	
No	786/16,809	82423.25	0.88 (0.74, 1.06)	
Body mass index				0.106
< 25 kg/m^2^	335/5,021	24985.33	1.17 (0.98, 1.39)	
25–29.9 kg/m^2^	330/5,903	28919.75	1.53 (1.27, 1.85)	
≥ 30 kg/m^2^	390/7,648	36330.75	1.28 (1.09, 1.51)	
Liver diseases				0.834
Yes	96/777	3428.42	1.13 (1.02, 1.25)	
No	969/17,815	86883.25	1.10 (0.78, 1.57)	

Age, gender, race, education level, ratio of family income to poverty, alcohol drinking status, smoking status, diabetes status, stroke status, coronary heart disease status, renal failure status, liver diseases status, high blood pressure status, myocardial infarction status, and cancer status were adjusted. In the subgroup analysis stratified by gender and race, the model is not adjusted for the stratification variable itself

### WWI, BMI and WC in predicting all-cause mortality

**Fig. 4:**
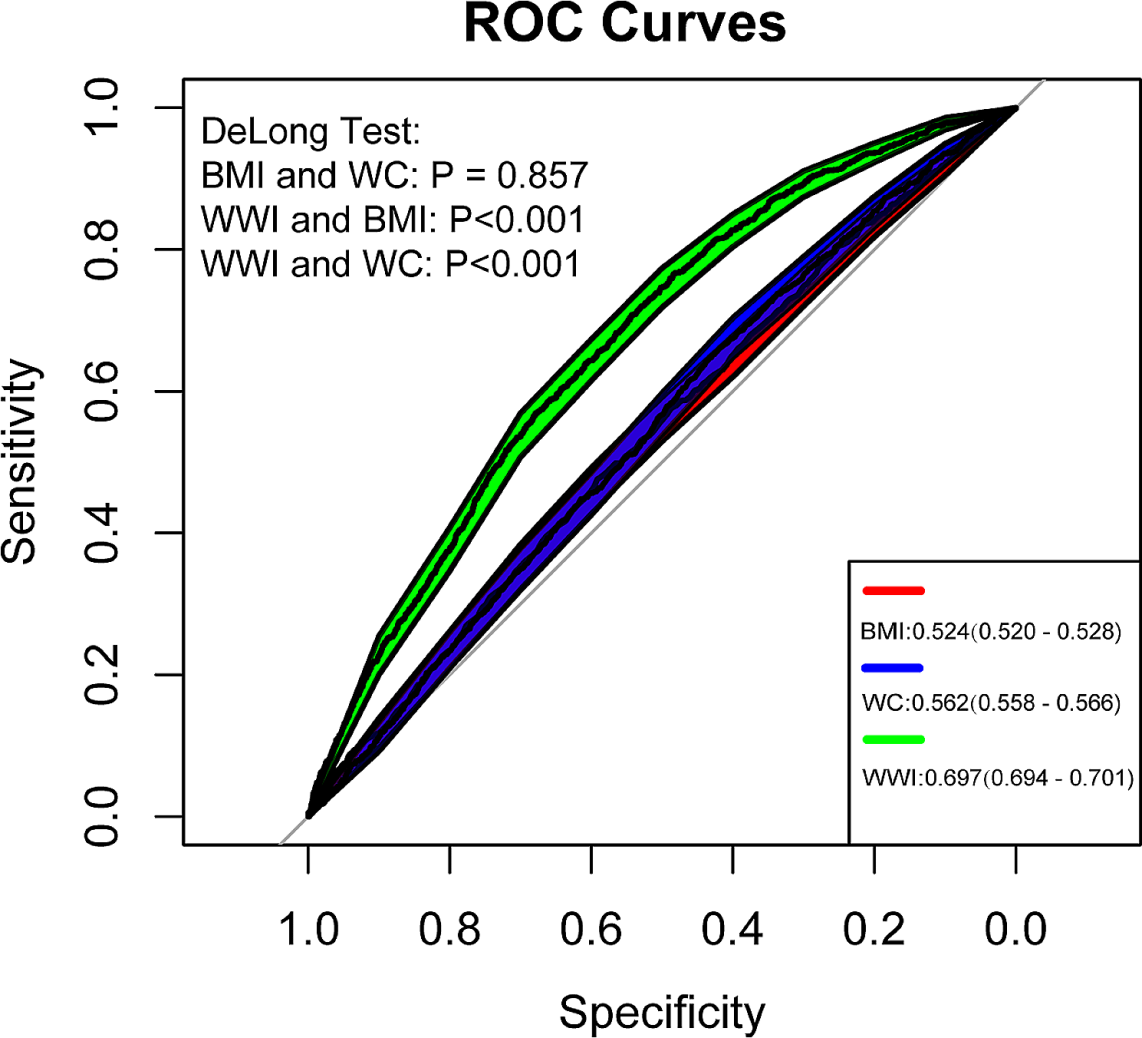
Receiver-operating characteristics (ROC) curves of weight-adjusted waist index, body mass index, and waist circumference for predicting all-cause mortality

Figure 4 demonstrates the predictive performance of WWI, BMI and WC on all-cause mortality. The AUCs for WWI, BMI and WC were 0.697, 0.524 and 0.562, respectively.

## Discussion

To our knowledge, this is the first extensive cohort study to explore the relationship between WWI and all-cause mortality, primarily focusing on non-Asian populations in the United States. This study provides a meticulous examination of the correlation between the WWI and all-cause mortality, involving comprehensive subgroup and sensitivity analyses, reaffirming the strength and consistency of our findings. Our study unveiled a U-shaped correlation between WWI and all-cause mortality, indicating a higher risk at both extremely low and high WWI values. In addition, our results demonstrate that WWI is more robustly associated with and predicts all-cause mortality than traditional measures of obesity such as BMI and WC, confirming its superiority in the study population prognostic ability.

Existing research highlights obesity as the leading lifestyle-related risk factor for premature mortality [40], underscoring the importance of evaluating obesity and identifying individuals at risk of metabolic disorders in clinical practice. Obesity assessment involves multiple anthropometric measures, with BMI being the most commonly utilized due to its regular measurement in primary care and hospital settings, as well as its straightforward classification into normal, overweight, and obese categories [41]. Despite its widespread use, BMI is not a highly accurate measure of body fat and composition, as it does not differentiate between lean body mass and fat mass. Additionally, body composition can differ among various demographic groups, including age, gender, and ethnicity, making it challenging to establish a universal age-sex-race index for defining obesity [42]. Research has demonstrated that Asians with lower BMI levels may face a greater risk of developing diabetes compared to individuals of European and American descent [43]. These findings clearly advocate for a more accurate and clinically useful measure of obesity.

Park et al. first proposed the WWI based on the classical approach of developing the height scaling index used in BMI and validated the predictive power for cardiovascular disease mortality and morbidity in a cohort of approximately 20,000 participants in Korea [21]. As the WWI was developed and validated using the same dataset, further validation of its reliability is still required by additional prospective studies [44]. In a 10-year cohort study of 1,863 Chinese older adults, Cai et al. validated a positive correlation between WWI and all-cause mortality. Their results indicated that WWI was a superior predictor of all-cause mortality compared to both BMI and WC [27]. Similarly, Ding et al. conducted a cohort study of more than 12,000 participants from southern China to examine the association between WWI and mortality [28], their study found a non-linear positive correlation between WWI with both all-cause mortality and cardiovascular mortality, with a significant increase in the risk of all-cause mortality when WWI was ≥ 11.2 cm/√kg. In our study, the predictive performance of WWI, BMI and WC for all-cause mortality was compared, and the results were expressed as AUC. The AUC for WWI, BMI and WC were 0.697, 0.524 and 0.562, respectively, and the results of the Delong test indicated that the predictive performance of WWI was significantly higher than that of BMI and WC (*P* < 0.001). These results further substantiated that WWI was a more effective predictor of all-cause mortality than BMI and WC. The AUC for WWI being notably higher than that of BMI and WC indicates that the WWI provides a more accurate prediction of mortality risk. Furthermore, our results revealed a U-shaped association between WWI and all-cause mortality in the US population, with a threshold effect analysis indicating that the risk of all-cause mortality is lowest at a WWI of 10.46 cm/√kg. These findings may have implications for using WWI to assess and manage obesity in specific populations to reduce the risk of death.

The relationship between obesity and increased risk of death in the general population is multifaceted and well-established [45]. However, it is crucial to take into account factors such as age, gender, and race when examining this association within a study population [46, 47]. Subgroup analyses indicate that non-Hispanic blacks demonstrate a distinct pattern compared to other races regarding the relationship between weight and all-cause mortality. Previous research suggests that this racial difference may be primarily attributed to variations in body size and composition [48]. Non-Hispanic Blacks of the same age, BMI, weight, and height tend to have longer limbs, greater musculoskeletal mass, and smaller trunk mass and waist circumference in comparison to individuals of other races [49]. Moreover, our analysis revealed a significant inverse association between weight and all-cause mortality in participants with cancers. Evidence suggests that tumors in obese patients may exhibit less aggressive characteristics than those in normal weight patients [50]. Additionally, the excess adipose tissue in obese patients can act as a nutritional reserve, providing a survival advantage during times of stress such as anti-cancer treatment [51]. However, given the potential for reverse causality, this association should be interpreted cautiously in clinical practice [52].

Our study has several noteworthy strengths, including the use of a complex multi-stage probability sampling design, which bolsters the reliability and representativeness of our findings, and a large sample size, allowing investigation into diverse population subgroups. However, it is important to recognize certain limitations and their potential bias. First, we lacked data on all covariates that might influence mortality, such as drug use. This limitation might introduce confounding bias, potentially underestimating or overestimating the true association between WWI and mortality. Despite this, we controlled for multiple significant confounders, helping to mitigate such bias. To further assess the impact that unmeasured confounders might have on our results, we calculated the E-value for the observed association between WWI and all-cause mortality (HR = 1.13, 95% CI: 1.03, 1.25). The E-value of 1.51 indicates that any unmeasured confounder would need to be associated with both WWI exposure and all-cause mortality by at least a factor of 1.51 to fully account for the observed risk ratio. The relatively high E-value suggests that our findings are robust, even in the presence of potential unmeasured confounding. Secondly, potential changes in participants’ body composition and environmental factors over the lengthy follow-up could introduce a misclassification bias, possibly underestimating the association if, for instance, those with higher initial WWI lost weight over the follow-up. The potential magnitude of this bias is challenging to determine due to the absence of repeated measures of these factors. Thus, while these limitations may affect the reliability of our findings, the robust study design and extensive analyses offer confidence in our results.

## Conclusion

This study is the first to examine the relationship between WWI and all-cause mortality in detail in a non-Asian population in the United States, revealing a U-shaped relationship between WWI and all-cause mortality, and WWI as a predictor of all-cause mortality beyond Traditional measures of obesity (such as BMI and WC) were excluded. These findings may help to better understand and predict the relationship between obesity and mortality, as well as provide support for effective obesity management strategies.

### Electronic supplementary material

Below is the link to the electronic supplementary material.

Supplementary Material 1

## Data Availability

The survey data are publicly available on the internet for data users and researchers throughout the world ( www.cdc.gov/nchs/nhanes/ ).

## Abbreviations

WWI: weight-adjusted waist circumference index
BMI: Body mass index
WC: waist circumference
NHANES: National Health and Nutrition Examination Survey
NCHS: National Center for Health Statistics
NDI: National Death Index
HRs: hazard ratios
CIs: confidence intervals
ROC: receiver operating characteristics
AUC: area under the ROC curve

## References

[1] Lingvay I, Sumithran P, Cohen RV, le Roux CW (2022). Obesity management as a primary treatment goal for type 2 diabetes: time to reframe the conversation. Lancet.

[2] Jaacks LM, Vandevijvere S, Pan A, McGowan CJ, Wallace C, Imamura F, Mozaffarian D, Swinburn B, Ezzati M (2019). The obesity transition: stages of the global epidemic. Lancet Diabetes Endocrinol.

[3] Network GBDC. Global burden of Disease Study 2015 (GBD 2015) obesity and overweight prevalence 1980–2015. In.: IHME Seattle; 2017.

[4] Fontaine KR, Redden DT, Wang C, Westfall AO, Allison DB (2003). Years of life lost due to obesity. JAMA.

[5] Whitlock G, Lewington S, Sherliker P, Clarke R, Emberson J, Halsey J, Qizilbash N, Collins R, Peto R (2009). Body-mass index and cause-specific mortality in 900 000 adults: collaborative analyses of 57 prospective studies. Lancet.

[6] Ortega FB, Lavie CJ, Blair SN (2016). Obesity and Cardiovascular Disease. Circ Res.

[7] Koliaki C, Liatis S, Kokkinos A (2019). Obesity and cardiovascular disease: revisiting an old relationship. Metabolism.

[8] Villarroya F, Cereijo R, Gavaldà-Navarro A, Villarroya J, Giralt M (2018). Inflammation of brown/beige adipose tissues in obesity and metabolic disease. J Intern Med.

[9] Xie R, Zhang Y, Yan T, Huang X, Xie S, Liu C, Liu M (2022). Relationship between nonalcoholic fatty liver disease and bone mineral density in adolescents. Med (Baltim).

[10] Silva MVF, Loures CMG, Alves LCV, de Souza LC, Borges KBG, Carvalho MDG (2019). Alzheimer’s disease: risk factors and potentially protective measures. J Biomed Sci.

[11] Lega IC, Lipscombe LL. Review: diabetes, obesity, and Cancer-Pathophysiology and Clinical implications. Endocr Rev 2020, 41(1). 10.1210/endrev/bnz014 31722374

[12] Bardou M, Rouland A, Martel M, Loffroy R, Barkun AN, Chapelle N (2022). Review article: obesity and colorectal cancer. Aliment Pharmacol Ther.

[13] Petrelli F, Cortellini A, Indini A, Tomasello G, Ghidini M, Nigro O, Salati M, Dottorini L, Iaculli A, Varricchio A (2021). Association of Obesity with Survival Outcomes in patients with Cancer: a systematic review and Meta-analysis. JAMA Netw Open.

[14] Ross R, Neeland IJ, Yamashita S, Shai I, Seidell J, Magni P, Santos RD, Arsenault B, Cuevas A, Hu FB (2020). Waist circumference as a vital sign in clinical practice: a Consensus Statement from the IAS and ICCR Working Group on visceral obesity. Nat Rev Endocrinol.

[15] Shieh A, Karlamangla AS, Karvonen-Guttierez C, Greendale GA. Menopause-related changes in body composition are associated with subsequent bone mineral density and fractures: Study of Women’s Health Across the Nation. J Bone Min Res 2022. 10.1002/jbmr.4759 PMC10023299 36542065

[16] Ma M, Liu X, Jia G, Geng B, Xia Y (2022). The association between body fat distribution and bone mineral density: evidence from the US population. BMC Endocr Disord.

[17] Okorodudu DO, Jumean MF, Montori VM, Romero-Corral A, Somers VK, Erwin PJ, Lopez-Jimenez F (2010). Diagnostic performance of body mass index to identify obesity as defined by body adiposity: a systematic review and meta-analysis. Int J Obes (Lond).

[18] Cornier MA, Després JP, Davis N, Grossniklaus DA, Klein S, Lamarche B, Lopez-Jimenez F, Rao G, St-Onge MP, Towfighi A (2011). Assessing adiposity: a scientific statement from the American Heart Association. Circulation.

[19] Pischon T, Boeing H, Hoffmann K, Bergmann M, Schulze MB, Overvad K, van der Schouw YT, Spencer E, Moons KG, Tjønneland A (2008). General and abdominal adiposity and risk of death in Europe. N Engl J Med.

[20] Jacobs EJ, Newton CC, Wang Y, Patel AV, McCullough ML, Campbell PT, Thun MJ, Gapstur SM (2010). Waist circumference and all-cause mortality in a large US cohort. Arch Intern Med.

[21] Park Y, Kim NH, Kwon TY, Kim SG (2018). A novel adiposity index as an integrated predictor of cardiometabolic disease morbidity and mortality. Sci Rep.

[22] Kim NH, Park Y, Kim NH, Kim SG (2021). Weight-adjusted waist index reflects fat and muscle mass in the opposite direction in older adults. Age Ageing.

[23] Cai S, Zhu T, Ding Y, Cheng B, Zhang A, Bao Q, Sun J, Li M, Liu X, Wang S (2023). The relationship between the weight-adjusted-waist index and left ventricular hypertrophy in Chinese hypertension adults. Hypertens Res.

[24] Li Q, Qie R, Qin P, Zhang D, Guo C, Zhou Q, Tian G, Liu D, Chen X, Liu L (2020). Association of weight-adjusted-waist index with incident hypertension: the rural Chinese cohort study. Nutr Metab Cardiovasc Dis.

[25] Qin Z, Chang K, Yang Q, Yu Q, Liao R, Su B (2022). The association between weight-adjusted-waist index and increased urinary albumin excretion in adults: a population-based study. Front Nutr.

[26] Zierfuss B, Höbaus C, Herz CT, Pesau G, Koppensteiner R, Schernthaner GH (2020). Predictive power of novel and established obesity indices for outcome in PAD during a five-year follow-up. Nutr Metab Cardiovasc Dis.

[27] Cai S, Zhou L, Zhang Y, Cheng B, Zhang A, Sun J, Li M, Su Y, Bao Q, Zhang Y (2022). Association of the weight-adjusted-Waist Index with risk of all-cause mortality: a 10-Year Follow-Up study. Front Nutr.

[28] Ding C, Shi Y, Li J, Li M, Hu L, Rao J, Liu L, Zhao P, Xie C, Zhan B (2022). Association of weight-adjusted-waist index with all-cause and cardiovascular mortality in China: a prospective cohort study. Nutr Metab Cardiovasc Dis.

[29] Zhang Y, Xie R, Ou J (2022). A U-shaped association between serum albumin with total triiodothyronine in adults. J Clin Lab Anal.

[30] Xie R, Liu Y, Wang J, Zhang C, Xiao M, Liu M, Zhang Y. Race and gender differences in the associations between Cadmium exposure and bone Mineral Density in US adults. Biol Trace Elem Res 2022. 10.1007/s12011-022-03521-y 36508128

[31] Qin Z, Du D, Li Y, Chang K, Yang Q, Zhang Z, Liao R, Su B (2022). The association between weight-adjusted-waist index and abdominal aortic calcification in adults aged ≥ 40 years: results from NHANES 2013–2014. Sci Rep.

[32] Ouyang Y, Quan Y, Guo C, Xie S, Liu C, Huang X, Huang X, Chen Y, Xiao X, Ma N (2022). Saturation effect of body Mass Index on Bone Mineral density in adolescents of different ages: a Population-based study. Front Endocrinol (Lausanne).

[33] Zhang YB, Chen C, Pan XF, Guo J, Li Y, Franco OH, Liu G, Pan A (2021). Associations of healthy lifestyle and socioeconomic status with mortality and incident cardiovascular disease: two prospective cohort studies. BMJ.

[34] Liu CA, Liu T, Ge YZ, Song MM, Ruan GT, Lin SQ, Xie HL, Shi JY, Zheng X, Chen Y (2023). Muscle distribution in relation to all-cause and cause-specific mortality in young and middle-aged adults. J Transl Med.

[35] Jayanama K, Theou O, Godin J, Mayo A, Cahill L, Rockwood K (2022). Relationship of body mass index with frailty and all-cause mortality among middle-aged and older adults. BMC Med.

[36] Xie R, Ning Z, Xiao M, Li L, Liu M, Zhang Y. Dietary inflammatory potential and biological aging among US adults: a population-based study. Aging Clin Exp Res 2023. 10.1007/s40520-023-02410-1 37186209

[37] Xie R, Zhang Y. Associations between dietary flavonoid intake with hepatic steatosis and fibrosis quantified by VCTE: evidence from NHANES and FNDDS. Nutr Metab Cardiovasc Dis 2023. 10.1016/j.numecd.2023.03.005 36964061

[38] Xie R, Xiao M, Li L, Ma N, Liu M, Huang X, Liu Q, Zhang Y (2022). Association between SII and hepatic steatosis and liver fibrosis: a population-based study. Front Immunol.

[39] Xie R, Zhang Y (2023). Association between 19 dietary fatty acids intake and rheumatoid arthritis: results of a nationwide survey. Prostaglandins Leukot Essent Fat Acids.

[40] Worldwide trends in (2017). Body-mass index, underweight, overweight, and obesity from 1975 to 2016: a pooled analysis of 2416 population-based measurement studies in 128·9 million children, adolescents, and adults. Lancet.

[41] Lennon H, Sperrin M, Badrick E, Renehan AG (2016). The obesity Paradox in Cancer: a review. Curr Oncol Rep.

[42] Arnold M, Leitzmann M, Freisling H, Bray F, Romieu I, Renehan A, Soerjomataram I (2016). Obesity and cancer: an update of the global impact. Cancer Epidemiol.

[43] Caleyachetty R, Barber TM, Mohammed NI, Cappuccio FP, Hardy R, Mathur R, Banerjee A, Gill P (2021). Ethnicity-specific BMI cutoffs for obesity based on type 2 diabetes risk in England: a population-based cohort study. Lancet Diabetes Endocrinol.

[44] Moons KG, Altman DG, Reitsma JB, Ioannidis JP, Macaskill P, Steyerberg EW, Vickers AJ, Ransohoff DF, Collins GS (2015). Transparent reporting of a multivariable prediction model for individual prognosis or diagnosis (TRIPOD): explanation and elaboration. Ann Intern Med.

[45] Hebebrand J, Holm JC, Woodward E, Baker JL, Blaak E, Durrer Schutz D, Farpour-Lambert NJ, Frühbeck G, Halford JGC, Lissner L (2017). A proposal of the European Association for the Study of Obesity to improve the ICD-11 diagnostic criteria for obesity based on the three dimensions Etiology, Degree of Adiposity and Health Risk. Obes Facts.

[46] Heymsfield SB, Peterson CM, Thomas DM, Heo M, Schuna JM, Hong S, Choi W (2014). Scaling of adult body weight to height across sex and race/ethnic groups: relevance to BMI. Am J Clin Nutr.

[47] Heymsfield SB, Peterson CM, Thomas DM, Heo M, Schuna JM (2016). Why are there race/ethnic differences in adult body mass index-adiposity relationships? A quantitative critical review. Obes Rev.

[48] Hruschka DJ, Hadley C, Brewis A (2014). Disentangling basal and accumulated body mass for cross-population comparisons. Am J Phys Anthropol.

[49] Hoffman DJ, Wang Z, Gallagher D, Heymsfield SB (2005). Comparison of visceral adipose tissue mass in adult African americans and whites. Obes Res.

[50] Hakimi AA, Furberg H, Zabor EC, Jacobsen A, Schultz N, Ciriello G, Mikklineni N, Fiegoli B, Kim PH, Voss MH (2013). An epidemiologic and genomic investigation into the obesity paradox in renal cell carcinoma. J Natl Cancer Inst.

[51] Demark-Wahnefried W, Platz EA, Ligibel JA, Blair CK, Courneya KS, Meyerhardt JA, Ganz PA, Rock CL, Schmitz KH, Wadden T (2012). The role of obesity in cancer survival and recurrence. Cancer Epidemiol Biomarkers Prev.

[52] Gelber RP, Kurth T, Manson JE, Buring JE, Gaziano JM (2007). Body mass index and mortality in men: evaluating the shape of the association. Int J Obes (Lond).

