# Supplementary material for: Association of weight-adjusted waist index with all-cause mortality among non-Asian individuals: a national population-based cohort study
Source: Nutr J. 2024 Jun 12;23:62. doi: 10.1186/s12937-024-00947-z (PMC11167926; doi:10.1186/s12937-024-00947-z)
Supplement: Supplementary file 1 — Supplementary Material 1 [file 12937_2024_947_MOESM1_ESM.docx]

| **Exposure** | **Model 1**  **HR (95% CI)** | **Model 2**  **HR (95% CI)** | **Model 3**  **HR (95% CI)** |
| --- | --- | --- | --- |
| WWI (continuous) | 2.05 (1.87, 2.24) | 1.36 (1.21, 1.53) | 1.27 (1.12, 1.44) |
| WWI (quartile) |  |  |  |
| Q1 | Reference | Reference | Reference |
| Q2 | 1.62 (1.17, 2.23) | 0.84 (0.61, 1.17) | 0.81 (0.58, 1.13) |
| Q3 | 2.91 (2.16, 3.91) | 1.07 (0.78, 1.46) | 0.98 (0.71, 1.35) |
| Q4 | 5.25 (3.97, 6.94) | 1.44 (1.05, 1.96) | 1.29 (0.94, 1.77) |
| P for trend | <0.001 | <0.001 | 0.004 |
| BMI (continuous) | 0.99 (0.98, 1.00) | 0.99 (0.98, 1.01) | 0.99 (0.98, 1.01) |
| BMI (quartile) |  |  |  |
| Q1 | Reference | Reference | Reference |
| Q2 | 0.94 (0.75, 1.18) | 0.68 (0.54, 0.85) | 0.71 (0.56, 0.89) |
| Q3 | 0.98 (0.79, 1.22) | 0.72 (0.58, 0.90) | 0.73 (0.58, 0.92) |
| Q4 | 0.82 (0.65, 1.04) | 0.77 (0.61, 0.98) | 0.79 (0.62, 1.01) |
| P for trend | 0.138 | 0.090 | 0.117 |
| WC (continuous) | 1.01 (1.01, 1.02) | 1.00 (1.00, 1.01) | 1.00 (1.00, 1.01) |
| WC (quartile) |  |  |  |
| Q1 | Reference | Reference | Reference |
| Q2 | 1.24 (0.97, 1.59) | 0.71 (0.55, 0.92) | 0.78 (0.60, 1.01) |
| Q3 | 1.51 (1.19, 1.93) | 0.69 (0.54, 0.88) | 0.73 (0.56, 0.95) |
| Q4 | 1.81 (1.43, 2.30) | 0.91 (0.72, 1.16) | 0.93 (0.73, 1.19) |
| P for trend | <0.001 | 0.938 | 0.954 |

**Table S1.** Association between WWI, BMI, WC, and all-cause mortality. (Participants without major CVD and cancers)

Participants with major CVD and cancers were excluded (Total excluded numbar: N=3,193; rspectively excluded number: major CVD: N=1,802, cancers: N=1,783).

Model 1: No covariates were adjusted. Model 2: Age, gender, race were adjusted. Model 3: Age, gender, race, education level, ratio of family income to poverty, alcohol drinking status, smoking status, renal failure status, myocardial infarction status, liver diseases status, and cancer status were adjusted.

Abbreviation: Q, quartile; WWI, weight-adjusted-waist index; BMI, body mass index; WC, waist circumference.

| **Exposure** | **Model 1**  **HR (95% CI)** | **Model 2**  **HR (95% CI)** | **Model 3**  **HR (95% CI)** |
| --- | --- | --- | --- |
| WWI (continuous) | 2.08 (1.92, 2.26) | 1.29 (1.16, 1.43) | 1.18 (1.06, 1.33) |
| WWI (quartile) |  |  |  |
| Q1 | Reference | Reference | Reference |
| Q2 | 1.88 (1.40, 2.53) | 0.85 (0.63, 1.14) | 0.81 (0.59, 1.11) |
| Q3 | 3.22 (2.45, 4.23) | 0.97 (0.73, 1.29) | 0.87 (0.64, 1.17) |
| Q4 | 5.75 (4.44, 7.46) | 1.29 (0.97, 1.72) | 1.12 (0.83, 1.51) |
| P for trend | <0.001 | <0.001 | 0.033 |
| BMI (continuous) | 0.99 (0.98, 1.00) | 0.99 (0.98, 1.00) | 0.99 (0.98, 1.00) |
| BMI (quartile) |  |  |  |
| Q1 | Reference | Reference | Reference |
| Q2 | 0.97 (0.80, 1.19) | 0.70 (0.57, 0.85) | 0.67 (0.54, 0.83) |
| Q3 | 0.96 (0.79, 1.18) | 0.75 (0.61, 0.91) | 0.73 (0.59, 0.90) |
| Q4 | 0.83 (0.67, 1.03) | 0.84 (0.68, 1.04) | 0.83 (0.67, 1.03) |
| P for trend | 0.087 | 0.247 | 0.235 |
| WC (continuous) | 1.01 (1.01, 1.02) | 1.00 (1.00, 1.01) | 1.00 (0.99, 1.01) |
| WC (quartile) |  |  |  |
| Q1 | Reference | Reference | Reference |
| Q2 | 1.20 (0.95, 1.50) | 0.67 (0.53, 0.84) | 0.69 (0.55, 0.88) |
| Q3 | 1.63 (1.32, 2.02) | 0.71 (0.57, 0.88) | 0.71 (0.57, 0.90) |
| Q4 | 1.70 (1.37, 2.10) | 0.87 (0.70, 1.08) | 0.85 (0.68, 1.07) |
| P for trend | <0.001 | 0.805 | 0.654 |

**Table S2.** Association between WWI, BMI, WC, and all-cause mortality. (Participants with a follow-up time > 2 years)

Participants with a follow-up time ≤ 2 years were excluded, N=2,677.

Model 1: No covariates were adjusted. Model 2: Age, gender, race were adjusted. Model 3: Age, gender, race, education level, ratio of family income to poverty, alcohol drinking status, smoking status, stroke status, coronary heart disease status, renal failure status, liver diseases status, myocardial infarction status, and cancer status were adjusted.

Abbreviation: Q, quartile; WWI, weight-adjusted-waist index; BMI, body mass index; WC, waist circumference.

**Table S3.** Association between WWI, BMI, WC, and all-cause mortality.

| **Exposure** | **number of participants/ number of events** | **Person-Years (years)** | **Model 1**  **HR (95% CI)** | **Model 2**  **HR (95% CI)** | **Model 3**  **HR (95% CI)** |
| --- | --- | --- | --- | --- | --- |
| WWI (continuous) | 1,065/18,592 | 90311.67 | 2.04 (1.90, 2.18) | 1.50 (1.37, 1.63) | 1.20 (1.09, 1.32) |
| WWI (quartile) |  |  |  |  |  |
| Q1 | 94/4,648 | 23937.33 | Reference | Reference | Reference |
| Q2 | 191/4,648 | 23028.08 | 2.13 (1.66, 2.72) | 1.20 (0.93, 1.55) | 1.09 (0.84, 1.42) |
| Q3 | 303/4,648 | 22280.33 | 3.51 (2.79, 4.43) | 1.46 (1.14, 1.87) | 1.11 (0.86, 1.44) |
| Q4 | 477/4,648 | 21065.92 | 5.93 (4.75, 7.40) | 2.10 (1.65, 2.68) | 1.36 (1.05, 1.77) |
| P for trend |  |  | <0.001 | <0.001 | 0.003 |
| BMI (continuous) | 1,065/18,592 | 90311.67 | 0.98 (0.97, 0.99) | 0.98 (0.97, 0.99) | 0.97 (0.96, 0.98) |
| BMI (quartile) |  |  |  |  |  |
| Q1 | 310/4,605 | 23465.08 | Reference | Reference | Reference |
| Q2 | 273/4,641 | 22805.42 | 0.89 (0.75, 1.04) | 0.66 (0.56, 0.78) | 0.60 (0.50, 0.71) |
| Q3 | 258/4,637 | 22320.25 | 0.85 (0.72, 1.00) | 0.65 (0.55, 0.77) | 0.56 (0.47, 0.67) |
| Q4 | 214/4,689 | 21645.08 | 0.73 (0.61, 0.87) | 0.67 (0.57, 0.80) | 0.51 (0.42, 0.62) |
| P for trend |  |  | <0.001 | <0.001 | <0.001 |
| WC (continuous) | 1,065/18,592 | 90311.67 | 1.01 (1.01, 1.01) | 1.00 (0.99, 1.00) | 0.99 (0.99, 0.99) |
| WC (quartile) |  |  |  |  |  |
| Q1 | 207/4,640 | 23672.17 | Reference | Reference | Reference |
| Q2 | 257/4,654 | 22812.75 | 1.27 (1.06, 1.53) | 0.77 (0.64, 0.93) | 0.78 (0.64, 0.95) |
| Q3 | 294/4,632 | 22208.67 | 1.51 (1.26, 1.80) | 0.72 (0.60, 0.87) | 0.64 (0.52, 0.77) |
| Q4 | 307/4,666 | 21618.08 | 1.62 (1.36, 1.93) | 0.83 (0.69, 1.00) | 0.62 (0.51, 0.75) |
| P for trend |  |  | <0.001 | 0.144 | <0.001 |

Age was used as a categorical variable (<40 years, 40-60 years, >60 years) to adjust the model.

Model 1: No covariates were adjusted. Model 2: Age, gender, race were adjusted. Model 3: Age, gender, race, education level, ratio of family income to poverty, alcohol drinking status, smoking status, stroke status, coronary heart disease status, renal failure status, liver diseases status, myocardial infarction status, and cancer status were adjusted.

Abbreviation: Q, quartile; WWI, weight-adjusted-waist index; BMI, body mass index; WC, waist circumference.
